# Supplementary material for: The genome sequence of the Alpine Milkvetch, Astragalus alpinus L. (Fabales: Fabaceae)
Source: Wellcome Open Res. 2025 Jul 25;10:366. [Version 1] doi: 10.12688/wellcomeopenres.24576.1 (PMC12398678; doi:10.12688/wellcomeopenres.24576.1)
Supplement: Peer review history

# Reviewer report: Reviewer response for version 1

**DOI:** 10.21956/wellcomeopenres.27085.r128949

**Published:** 2025-09-10

**Authors:** Yedomon Ange Bovys Zoclanclounon

**Affiliations:** 1 Rothamsted Research, Harpenden, England, UK

The manuscript presents the sequencing and assembly of the nuclear, mitochondrial, and plastid genomes of *Astragalus alpinus*. This foundational genomic resource constitutes an important step toward understanding the genetic basis of the species’ unique adaptations to Arctic–alpine environments and addressing its ongoing conservation challenges.

 Overall, the manuscript is clearly written, well structured, and the results are appropriately supported by the analyses presented. The reported ~100× Hi-C coverage provides a robust foundation for a reliable chromosome-scale assembly of the species. The work is timely and of relevance to both the plant genomics and conservation biology communities.

 My minor comments are as follows:

1. **Sequence data**: The HiFi read size and number of reads are reported as 0.00 Gb from 0.00 million reads, which appears to be an omission. A check of the European Nucleotide Archive ( https://www.ebi.ac.uk/ena/browser/view/ERX12134038) indicates that only the BAM file has been deposited, while the corresponding FASTQ file is not yet available. Clarification and correction are needed.
2. **Transcriptome data**: Although a transcriptome dataset was generated, its use is not discussed in the manuscript (e.g., mapping rate onto the haplotype 1 assembly). In addition, the coverage is relatively low (~12.24×) and limited to a single tissue (leaf). While the scope of this report is not comparative genomics, increasing RNA-seq coverage across multiple tissues could provide valuable insights into the genetic basis of *A. alpinus*’ ecological adaptations, and this possibility could be noted as a future perspective.

Are sufficient details of methods and materials provided to allow replication by others?

Yes

Is the rationale for creating the dataset(s) clearly described?

Yes

Are the datasets clearly presented in a useable and accessible format?

Yes

Are the protocols appropriate and is the work technically sound?

Yes

Reviewer Expertise:

genome assembly, genome annotation, genome evolution, comparative genomics, multi-omics integration

I confirm that I have read this submission and believe that I have an appropriate level of expertise to confirm that it is of an acceptable scientific standard.

# Reviewer report: Reviewer response for version 1

**DOI:** 10.21956/wellcomeopenres.27085.r128950

**Published:** 2025-08-30

**Authors:** Oliver Rupp

**Affiliations:** 1 Justus-Liebig-University Giessen, giessen, Germany

This article presents the sequencing and assembly of the *Astragalus alpinus* genome.

 The data creation and data analyses follow the state-of-the art methods for plant genome assemblies, including PacBio Hifi and Hi-C sequencing, hifiasm assembly followed by automatic and manual assembly curation. The assembly quality was assessed by k-mer and orthology based methods like Merqury.FK or BUSCO.

 The methods are clearly presented and the quality of the data is shown.

 The pipeline and data are available at public databases and a gene annotation will be performed.

 Overall no major issues are to report.

 The genome sequence report paragraph states "... generating 0.00 Gb (gigabases) from 0.00 million reads ...", this should be checked.

Are sufficient details of methods and materials provided to allow replication by others?

Yes

Is the rationale for creating the dataset(s) clearly described?

Yes

Are the datasets clearly presented in a useable and accessible format?

Yes

Are the protocols appropriate and is the work technically sound?

Yes

Reviewer Expertise:

Bioinformatics, genome assembly, RNAseq analyses

I confirm that I have read this submission and believe that I have an appropriate level of expertise to confirm that it is of an acceptable scientific standard.

# Reviewer report: Reviewer response for version 1

**DOI:** 10.21956/wellcomeopenres.27085.r128947

**Published:** 2025-08-23

**Authors:** Qionghou Li

**Affiliations:** 1 Nanjing Agricultural University, Nanjing, Jiangsu, China

Ruhsam et al. present the first chromosome-scale genome assembly of *Astragalus alpinus*. Using PacBio HiFi reads with Hi-C scaffolding, author report 96.8% BUSCO completeness and annotate the assembly with RNA-seq data. Overall, the manuscript reports two high-quality, haplotype-resolved chromosome-level assemblies. I have only some minor concerns:

1. Since the authors evaluated the assembly with Merqury. I recommend adding QV, k-mer completeness, and genome BUSCO completeness to Table 2.
2. The genome annotation was also performed, please also include in Table 2 the number of protein-coding genes, the number of transcripts, and gene-level BUSCO completeness.
3. Although the study reported PacBio HiFi sequencing, both Table 1 and the sentence—“The genome of a specimen of *Astragalus alpinus* was sequenced using Pacific Biosciences single-molecule HiFi long reads, generating 0.00 Gb (gigabases) from 0.00 million reads”—report 0 data yield. Inspecting the ENA (EMBL-EBI) submission (ERR12760829: https://www.ebi.ac.uk/ena/browser/view/ERR12760829) shows the HiFi data are provided as BAM files, for which ENA does not automatically compute read counts. The authors should therefore report the total HiFi read count and total bases manually. If that is inconvenient, I suggest converting BAM to FASTQ with PacBio’s bam2fastx ( https://github.com/PacificBiosciences/pbtk) and then calculating the statistics with seqkit, and adding these values to draft.

Are sufficient details of methods and materials provided to allow replication by others?

Yes

Is the rationale for creating the dataset(s) clearly described?

Yes

Are the datasets clearly presented in a useable and accessible format?

Yes

Are the protocols appropriate and is the work technically sound?

Yes

Reviewer Expertise:

genome assembly, genome evolution, comparative genomics

I confirm that I have read this submission and believe that I have an appropriate level of expertise to confirm that it is of an acceptable scientific standard.
